# Earthquake exposures and mental health outcomes in children and adolescents from Phulpingdanda village, Nepal: a cross-sectional study

**DOI:** 10.1186/s13034-018-0257-9

**Published:** 2018-12-20

**Authors:** Jessica S. Schwind, Clara B. Formby, Susan L. Santangelo, Stephanie A. Norman, Rebecca Brown, Rebecca Hoffman Frances, Elisabeth Koss, Dibesh Karmacharya

**Affiliations:** 1 Department of Biostatistics, Epidemiology, and Environmental Health Sciences, Jiann-Ping Hsu College of Public Health, Georgia Southern University, P.O. Box 8015, Statesboro, Georgia 30460 USA; 2 Medical College of Georgia, Augusta University, 1120 15th Street, Augusta, GA 30912 USA; 3 Maine Medical Research Institute, 509 Forest Ave, Suite 200B, Portland, ME 04101 USA; 4 Tufts University School of Medicine, 136 Harrison Avenue, Boston, MA 02111 USA; 5 St. Joseph’s College of Maine, 278 Whites Bridge Road, Standish, ME 04084 USA; 6 Maine Behavioral Healthcare, 165 Lancaster Street, Portland, ME 04101 USA; 7 Independent Researcher, Oakland, CA 94609 USA; 8 Center for Molecular Dynamics Nepal, GPO Box 21049, Kathmandu, Nepal

**Keywords:** Earthquake, Resilience, Depression, PTSD, Nepal, Children, Adolescents, Natural disaster

## Abstract

**Background:**

Mental health issues can reach epidemic proportions in developed countries after natural disasters, but research is needed to better understand the impact on children and adolescents in developing nations.

**Methods:**

A cross-sectional study was performed to examine the relationship between earthquake exposures and depression, PTSD, and resilience among children and adolescents in Phulpingdanda village in Nepal, 1 year after the 2015 earthquakes, using the Depression Self-Rating Scale for Children, Child PTSD Symptom Scale, and the Child and Youth Resilience Measure, respectively. To quantify exposure, a basic demographic and household questionnaire, including an earthquake exposure assessment tool for children and adolescents, was created.

**Results:**

Of the 62 respondents interviewed, 3.23% and 4.84% displayed symptomatology of depression and PTSD. A large number of respondents interviewed scored high for resiliency (80.65%). All 62 respondents were displaced from their household and witnessed severe damage of both their homes and village. The number of earthquake exposures had a strong, positive correlation with PTSD symptomatology.

**Conclusions:**

Although the number of respondents who showed signs of depression and PTSD symptomatology was lower than anticipated, resilience scores were considerably higher. Future research should explore which protective factors may contribute to high resiliency in Nepali children and adolescents.

**Electronic supplementary material:**

The online version of this article (10.1186/s13034-018-0257-9) contains supplementary material, which is available to authorized users.

## Background

After a natural disaster, the initial actions of an emergency response team are to provide for basic human needs and begin the search and rescue process. The recovery phase can begin once there is no longer an immediate threat to human life [1]. One of the most neglected factors, but arguably one with the longest impact in emergency response, is the need to address mental health issues that arise in the wake of disaster [2]. There is ample research on the adverse effects natural disasters can have on mental health [3–5]. Earthquakes, a common natural disaster, are the focus of many studies on post-disaster mental health complications, particularly depression and posttraumatic stress disorder (PTSD) [6–9]. Though much attention has been given to adverse mental health outcomes, an emerging field of resilience research is focused on how a population can successfully adapt to the effects of trauma [10, 11]. According to The World Health Organization (WHO), an estimated 20% of any given population is at increased risk of developing mental health problems after a disaster [12]. Although every population will experience some increase in risk, not all populations respond to tragedy in the same way [13]. Developed nations and economically advantaged communities have the ability to respond to disaster somewhat efficiently, which may lessen the negative impact on health. However, economic instability, political and economic corruption, and chronic lack of basic resources can hinder the ability of developing countries to respond to and recover from natural disasters [14–16].

On April 25, 2015, an earthquake with a magnitude of 7.8 struck much of South Asia, majorly impacting the country of Nepal. The initial earthquake and subsequent aftershocks killed more than 8890 people and injured over 22,300. Several aftershocks, including a shock with a magnitude of 7.3 in May, created further damage and mortality. In Nepal alone, around 2.8 million people were displaced and forced to live indefinitely in makeshift tent villages [2]. Studying the aftermath in hard-hit countries like Nepal after the 2015 earthquakes is key to a better understanding of the mental health outcomes that can result from disaster in an already struggling nation. Preliminary studies have been published, predominantly focused on adult populations, from data collected 4–6 months after the earthquakes [17–19]. However, prior research has shown up to 25% of PTSD cases are not observed clinically until 12 months after the event [20].

Children who experience natural disasters are not immune to the risk of developing mental health problems, and may be more adversely affected by the trauma [21]. Past studies have shown mental health problems of children in developed countries after natural disasters can reach epidemic proportions and can last for long periods of time, potentially causing lifelong disabilities [7, 21]. Given that children and adolescents, as well as developing countries in general, are especially vulnerable to adverse mental health outcomes after natural disasters, children in Nepal may face an even greater threat of adverse mental health problems after the 2015 earthquakes. In the past, the primary focus of mental health research for children in Nepal has been on the impact of years of political turmoil and the use of child soldiers [22–24]. Therefore, the objective of this study was to examine the relationship between earthquake exposures and resilience, as well as symptoms of depression and PTSD, among children and adolescents (ages 8–17) in the Sindhupalchowk district of Nepal 1 year after the 2015 earthquakes.

## Methods

### Study population

Mental health and earthquake exposure assessments were administered approximately 12 months after the earthquakes in the village of Phulpingdanda in the Sindhupalchowk district. Located approximately 100 km from the initial earthquake’s epicenter, the Sindhupalchowk district was one of the worst affected, due to high magnitude aftershocks and geographical remoteness. Potential households were identified by a village spokesperson and invited to participate in the study. All respondents within each household, ages 8–85, who had experienced the earthquakes were invited to interview. However, in order to better understand the impact on different age groups, data were analyzed separately for children and adolescents, age 8–17, and adults, age 18–85.

### Procedure

All subjects who met the inclusion criteria were assessed with Nepali versions of the following instruments: the Earthquake Exposure Assessment Tool for Children and Adolescents (EEAT), the Depression Self-Rating Scale for Children (DSRSC), the Child PTSD Symptom Scale (CPSS), the Child and Youth Resilience Measure (CYRM), and basic demographic and household questionnaires. Because validated instruments were not available in the Nepali language, a bilingual legal and public health expert in Nepal translated the English screening tools to be used. An outside translator established the equivalence of the English and Nepali versions through blind back translation to ensure proper translation.

Co-investigators and interviewers thoroughly reviewed the entire questionnaire packet. A village spokesperson was established to discuss the study and solicit participation from villagers. All data were collected over a 2-week period. Two Nepali interviewers, fluent in both the local language and English, and with previous field experience, went through intensive training to improve interviewing skills and reduce interviewer bias. Interviewers followed a structured interview format with each participant and read all options listed for each question. Given the subjects’ age and possible literacy issues, the assent and consent form was read aloud by the interviewers to each respondent and their parent or guardian in a private location to help ensure a full understanding of the study. Respondents were interviewed and asked to complete the questionnaire only after assent was provided by the child or adolescent and parent or guardian consent was given. Special attention was given to subject privacy, cultural sensitivity, and local traditions. Study investigators supervised subject interviewing and checked data for completeness and formatting. All subjects who tested positive for symptoms of severe depression and/or PTSD were provided the contact information of a clinical practitioner for follow-up intervention. Ethical approval was obtained from the Nepal Health Research Council (Reg. No. 250/2015), the Augusta University Institutional Review Board (850,582), and the Maine Medical Center Research Institute Institutional Review Board (958976).

### Measures

*Demographic characteristics* were age and gender of the respondent. The following variables were also collected from the primary adult responder in the household: marital status of household (married/other), household identified religion (Hindu/other), household identified ethnic group (Upper caste/Relatively advantaged Janajatis/Disadvantaged Janajatis/Other), highest level of education of primary respondent (primary education or greater/illiterate or none), household identified occupation (farmer/other), approximate monthly household income in Nepali rupees (≤ 10 k/> 10 k), and number of members in household (≤ 3/4 to 5/≥ 6).

*Earthquake exposures* were measured through the Earthquake Exposure Assessment Tool for Children and Adolescents (EEAT), which was modified from the Hurricane Exposure Assessment Tool for Children and Adolescents to assess the number and type of earthquake exposures experienced (Additional file 1). The Hurricane Exposure Assessment for Children and Adolescents was originally developed for post-disaster screenings after Hurricane Katrina in 2005 and has since shown reliability in several different hurricane studies [25]. Past studies on earthquake exposures in children focused on potentially traumatic events that could have occurred during the earthquake based on either a modified assessment tool or general list of common traumatic events during a natural disaster [9, 26, 27]. A total EEAT score was calculated by summing the number of earthquake exposures for each child.

*Depression* was measured using the Depression Self-Rating Scale for Children (DSRSC). The DSRSC was created to assess moderate to severe depression in children aged 6–17, based on children’s answers to 18 easily understood questions. Answers were collected about the child’s feelings during the past week and recorded as occurring “mostly”, “sometimes”, or “never” [28]. Based on a maximum score of 36, preliminary studies recommended a cutoff score of 15, which was used for the sake of comparison to several cross-cultural studies [28–30]. However, a study in Nepali children suggests a cutoff score of greater than or equal to 14 demonstrated a sensitivity of 0.71 and specificity of 0.81 [31]. A score of greater than or equal to 14 was used for the present study.

*Post*-*traumatic stress disorder* was measured using the Child PTSD Symptom Scale (CPSS). The CPSS was designed to assess varying degrees of symptoms of a PTSD diagnosis in children aged 8–18. Part one has one question for each of the 17 possible symptoms relevant to a diagnosis of PTSD. Scores range from 0 to 51 based on a 4-point Likert scale [32]. Preliminary research on the psychometric properties of the CPSS suggested that a score greater than or equal to 11 was indicative of a PTSD diagnosis [32–34]. However, more recent literature suggested that a score of 20 or 21.5 or greater had better specificity for a diagnosis of PTSD [31, 35, 36]. A score of greater than or equal to 20 was used for the present study.

*Resilience* was measured using the shorter Child and Youth Resilience Measure (CYRM). The original 28-question CYRM had been reduced to a 12-question assessment scored on a 3-point Likert scale with higher scores indicating higher resilience. Though shorter, the CYRM-12 has proved to still be a valid assessment of resilient characteristics in children and adolescents [37, 38].

### Statistical analysis

Numbers and percentages of prevalent cases were determined based on the recommended scorings for the above screening tools. Descriptive statistics were used to determine associations between sociodemographic variables and earthquake exposures to mental health outcomes. Two sample *t*-tests with equal variances were used to determine associations between mental health outcome scores and two-group categorical independent variables, while an ANOVA test was used if there were more than two groups. Spearman correlation coefficients were calculated to help evaluate the relationship between depression and PTSD with resilience. Simple linear regression and Spearman correlation coefficients were calculated to assess the relationship of depression, PTSD, and resilience with earthquake exposures. All statistical analyses were conducted using STATA Version 15.0 (Stata Corp., College Station, TX) statistical software and performed on de-identified data. Statistical significance was set at p ≤ 0.05 (two-tailed).

## Results

After one participant was dropped due to missing age information, the final sample included 62 respondents (Table 1). Of the 62 children and adolescents interviewed, 27 (43.55%) were male and 35 (56.45%) were female. The average age of the sample was 12.5 years old (SD = 2.33). The majority of households identified as married (88.71%), Hindu (87.10%) and relied primarily on income from agricultural sources (85.48%). The sample population represented a variety of ethnicities and household sizes. These children experienced an average of 6.87 (SD = 1.52) earthquake exposures (Table 2). All respondents experienced badly damaged or destroyed homes and neighborhoods, but no respondents were separated from their parents or primary caretaker.

**Table 1:** Children and adolescent study population demographics

Characteristic	n (%)
Respondents’ age (mean, SD)	12.5, 2.33
Respondents’ gender	
Male	27 (43.55)
Female	35 (56.45)
Household marital status	
Married	55 (88.71)
Other	7 (11.29)
Household identified religion	
Hindu	54 (87.10)
Other	8 (12.90)
Household identified ethnicity	
Upper caste	25 (40.32)
Relatively advantaged Janajatis	16 (25.81)
Disadvantaged Janajatis	10 (16.13)
Other	11 (17.74)
Highest level of education in household	
Illiterate/none/no response	37 (59.68)
Primary or higher	25 (40.32)
Household identified occupation	
Farmer	53 (85.48)
Other	9 (14.52)
Monthly household income (Nepali rupees)	
≤ 10 k or no response	33 (53.23)
> 10 k	29 (46.77)
Household size	
≤ 3	8 (12.90)
4–5	30 (48.39)
≥ 6	24 (38.71)

**Table 2:** Earthquake exposure assessment tool for children and adolescents

Overall	Mean	SD	Range
n = 62	6.87	1.52	(4–11)

Statement	Yes	No
	n (%)	n (%)
Child seriously injured	3 (4.84)	59 (95.16)
Family member/friend seriously injured or killed	17 (27.42)	45 (72.58)
Witnessed injury or death	6 (9.68)	56 (90.32)
Was separated from parents or primary caretaker(s)	0 (0)	62 (100)
*Home destroyed, badly damaged by earthquakes*	62 (100)	0 (0)
Condition of home unknown	25 (40.32)	37 (59.68)
*Saw neighborhood destroyed or badly damaged*	62 (100)	0 (0)
Saw other areas destroyed or badly damaged	58 (93.55)	4 (6.45)
Pet: separated from, lost, hurt or killed	1 (1.61)	61 (98.39)
*Belongings, clothes/toys destroyed by earthquake*	46 (74.19)	16 (25.81)
Condition of belongings unknown	22 (35.48)	40 (64.52)
Trapped/difficulty evacuating	6 (9.68)	56 (90.32)
Isolated	1 (1.61)	61 (98.39)
In other crowded shelter	20 (32.26)	42 (67.74)
Exposed to violence or looting	1 (1.61)	61 (98.39)
Displaced from home; length of time in days (mean, SD)	64.43, 85.57	
*Number of shelter/displacement centers (mean, SD)*	1.53, 1.28	
Currently in shelter/displacement center	56 (90.32)	6 (9.68)
*Transferred to new school because of earthquakes*	5 (8.06)	57 (91.94)
Length of time in new school (weeks)	1.79, 7.96	
Helped in rescue/recovery efforts	18 (29.03)	44 (70.97)
Family member served as rescue/recovery worker	31 (50.00)	31 (50.00)
*Parent currently unemployed*	1 (1.61)	61 (98.39)
Before earthquakes	2 (3.23)	60 (96.77)
Because of earthquakes	2 (3.23)	60 (96.77)
Previous earthquake experience	6 (9.68)	56 (90.32)
Past major loss or trauma	2 (3.23)	60 (96.77)
Additional loss (livestock)	20 (32.26)	42 (67.74)

Based on suggested cutoff points, 3.23% (n = 2) of the interviewed children displayed depression symptomatology, 4.84% (n = 3) displayed PTSD symptomatology, and 80.65% (n = 50) showed signs of high resilience (Table 3). However, the negative correlation between depression and resilience and between PTSD and resilience were not statistically significant. Children aged 8–12 were found to have lower than expected scores for depressive symptomatology when compared to older adolescents (Table 4). Children from households identified as being from an upper caste or other ethnicity were found to have higher scores for depressive symptomatology compared to the Janajatis, Nepal’s indigenous people.

**Table 3:** Mental health outcomes in children and adolescents in Phulpingdanda

Clinical outcome			n (%)
Depression symptomatology			
No (< 14)			60 (96.77)
Yes (≥ 14)			2 (3.23)
(Mean, SD, range)	6.61	3.54	0–20
PTSD symptomatology			
No (< 20)			59 (95.16)
Yes (≥ 20)			3 (4.84)
(Mean, SD, range)	8.16	5.72	0–28
Resilience symptomatology			
No (< 30)			12 (19.35)
Yes (≥ 30)			50 (80.65)
(Mean, SD, range)	31.18	2.52	22–35

Spearman’s correlation: depression vs. resilience, ρ = (− 0.2113), p-value = 0.0992

Spearman’s correlation: PTSD vs. resilience, ρ = (− 0.1817), p-value = 0.1576

**Table 4:** Demographic and socioeconomic factors associated with mental health outcomes

Characteristic	Depression (mean ± SD)	PTSD (mean ± SD)	Resilience (mean ± SD)
Child’s age			
8–12	5.97 ± 3.53*	7.64 ± 5.86	30.83 ± 2.26
13–17	7.50 ± 3.42	8.88 ± 5.56	31.65 ± 2.81
Child’s gender			
Male	5.81 ± 2.88	7.85 ± 4.75	30.78 ± 2.39
Female	7.23 ± 3.90	8.40 ± 6.43	31.49 ± 2.61
Household’s marital status			
Married	6.56 ± 3.50	8.18 ± 6.00	31.05 ± 2.61
Other	7.00 ± 4.12	8.00 ± 2.89	32.14 ± 1.46
Household identified religion			
Hindu	6.61 ± 3.59	8.46 ± 5.90	31.15 ± 2.62
Other	6.23 ± 3.38	6.13 ± 4.01	31.38 ± 1.85
Household identified ethnicity			
Upper caste	7.36 ± 3.23*	9.52 ± 5.31	31.08 ± 2.94
Relatively advantaged Janajatis	4.48 ± 2.38	5.25 ± 3.75	31.38 ± 2.22
Disadvantaged Janajatis	6.80 ± 3.01	7.00 ± 4.52	31.60 ± 1.84
Other	7.91 ± 4.93	10.36 ± 8.24	30.73 ± 2.65
Highest level of education in household			
Illiterate/none/no response	6.78 ± 3.95	8.24 ± 6.34	31.14 ± 2.45
Primary or higher	6.36 ± 2.90	8.04 ± 4.80	31.24 ± 2.67
Household identified occupation			
Farmer	6.58 ± 3.17	7.83 ± 5.29	31.38 ± 2.55
Other	6.77 ± 5.49	10.11 ± 7.91	30.00 ± 2.06
Monthly household income (Nepali rupees)			
≤ 10 k or no response	6.85 ± 3.94	8.76 ± 5.73	31.33 ± 2.13
> 10 k	6.34 ± 3.07	7.48 ± 5.74	31.00 ± 2.93
Household size			
≤ 3	6.50 ± 2.93	7.13 ± 3.68	31.88 ± 2.03
4–5	6.83 ± 3.84	8.73 ± 6.60	31.50 ± 2.24
≥ 6	6.38 ± 3.45	7.79 ± 5.18	30.54 ± 2.92

*p-value < 0.05

When examining the relationship between earthquake exposures and mental health outcomes, children and adolescents with a family member or friend who was seriously injured or killed had significantly higher scores for both depressive and PTSD symptomatology relative to children without this exposure (Table 5). When examining the association between earthquake exposure scores and mental health outcomes, both depressive and PTSD symptoms were positively correlated with earthquake exposures (Table 6). However, PTSD symptoms had a stronger, positive correlation with earthquake exposures than did depressive symptoms.

**Table 5:** Association between earthquake exposures and mental health outcomes in children and adolescents

Statement	Depression (mean ± SD)	PTSD (mean ± SD)	Resilience (mean ± SD)
Child seriously injured			
Yes	4.67 ± 3.79	11.00 ± 6.00	31.33 ± 0.58
No	6.71 ± 3.53	8.02 ± 5.72	31.17 ± 2.58
Family member/friend seriously injured or killed			
Yes	8.59 ± 4.30*	11.76 ± 6.43*	31.00 ± 2.06
No	5.87 ± 2.93	6.80 ± 4.85	31.24 ± 2.69
Witnessed injury or death			
Yes	7.67 ± 2.66	11.17 ± 1.83	31.00 ± 2.76
No	6.50 ± 3.62	7.84 ± 5.91	31.20 ± 2.52
Was separated from parents or primary caretaker(s)			
Yes	–	–	–
No	–	–	–
Home destroyed, badly damaged by earthquakes			
Yes	–	–	–
No	–	–	–
Saw neighborhood destroyed or badly damaged			
Yes	–	–	–
No	–	–	–
Pet: separated from, lost, hurt or killed			
Yes	12.00 ± N/A	15.00 ± N/A	34.00 ± N/A
No	6.52 ± 3.50	8.05 ± 5.70	31.13 ± 2.51
Belongings, clothes/toys destroyed by earthquake			
Yes	6.43 ± 3.25	8.70 ± 5.29	31.48 ± 2.16
No	7.13 ± 4.35	6.63 ± 6.77	30.31 ± 3.28
Trapped/difficulty evacuating			
Yes	7.17 ± 3.37	8.33 ± 7.31	31.50 ± 2.43
No	6.55 ± 3.58	8.14 ± 5.61	31.14 ± 2.55
Isolated			
Yes	9.00 ± N/A	10.00 ± N/A	29.00 ± N/A
No	6.57 ± 3.56	8.13 ± 5.76	31.21 ± 2.52
In other crowded shelter			
Yes	7.70 ± 3.91	8.05 ± 6.64	31.25 ± 2.36
No	6.10 ± 3.27	8.21 ± 5.32	31.14 ± 2.62
Exposed to violence or looting			
Yes	9.00 ± N/A	5.00 ± N/A	30.00 ± N/A
No	6.57 ± 3.56	8.21 ± 5.76	31.20 ± 2.54
Displaced from home			
Yes	–	–	–
No	–	–	–
Experienced shelter/displacement centers			
Yes	6.73 ± 3.64	8.02 ± 5.61	31.09 ± 2.57
No	5.50 ± 2.43	9.50 ± 7.12	32.00 ± 1.90
Transferred to new school because of earthquakes			
Yes	5.80 ± 4.27	10.20 ± 6.38	32.80 ± 1.30
No	6.68 ± 3.51	7.98 ± 5.69	31.04 ± 2.56
Helped in rescue/recovery efforts			
Yes	6.11 ± 3.55	8.67 ± 5.32	31.89 ± 1.81
No	6.82 ± 3.56	7.95 ± 5.93	30.89 ± 2.72
Family member served as rescue/recovery worker			
Yes	6.42 ± 3.51	8.55 ± 4.77	31.29 ± 2.77
No	6.81 ± 3.62	7.77 ± 6.60	31.06 ± 2.28
Parent currently unemployed			
Yes	11.00 ± N/A	9.00 ± N/A	31.00 ± N/A
No	6.54 ± 3.52	8.15 ± 5.77	31.18 ± 2.54
Previous earthquake experience			
Yes	5.83 ± 4.12	6.33 ± 5.54	31.67 ± 2.16
No	6.70 ± 3.51	8.36 ± 5.76	31.13 ± 2.57
Past major loss or trauma			
Yes	9.00 ± 1.41	9.00 ± 4.24	32.50 ± 2.12
No	6.53 ± 3.57	8.13 ± 5.79	31.13 ± 2.53
Additional loss (livestock)			
Yes	6.45 ± 3.28	8.65 ± 5.18	31.95 ± 1.76
No	6.69 ± 3.69	7.93 ± 6.01	30.81 ± 2.75

*p-value < 0.05

**Table 6:** Linear regression and spearman correlation results when examining the association between earthquake exposure scores and mental health outcomes

Mental health outcomes	Linear regression		Spearman correlation	
	R^2^	p-value	ρ	p-value
Depression symptomatology	0.023	0.238	0.211	0.100
PTSD symptomatology	0.071	0.036	0.335	0.008
Resilience symptomatology	0.052	0.074	0.234	0.067

## Discussion

Based on the Earthquake Exposure Assessment Tool, all children and adolescents interviewed witnessed severe damage or destruction of both their homes and neighborhoods. Although every child interviewed was also displaced from the home for some period of time, the number of respondents displaying symptoms of depression and PTSD were lower than expected. An overwhelming 80.65% of the children interviewed scored considerably high for resiliency. However resiliency was negatively but non-significantly correlated with both depression and PTSD. The small sample size was likely the reason for the non-significant correlation. Younger respondents had lower depression scores than older children, while respondents from upper caste households or ‘other’ ethnicity/castes had higher depression scores than the Janajatis. Respondents who experienced a family member or friend seriously injured or killed had higher scores on both depression and PTSD symptomatology.

Of the 62 children and adolescents interviewed, the overall number of respondents displaying symptomatology of any adverse mental health outcome was 4.84% (n = 3). This is in contrast to a 2012 WHO study that examined data from various countries and exposure levels that estimated that 15–20% of the population is expected to demonstrate mild to moderate adverse mental health outcomes after an emergency [12]. Based on a review of psychological effects of earthquakes on children and adolescents, the prevalence of depression and PTSD varies greatly from study to study. The results of the current study were lower for both outcomes based on the ranges observed [39]. The lower than expected depression scores seen in children aged 8–12 were consistent with findings in a previous earthquake study that suggested more severe depressive and PTSD symptoms were seen in older adolescents [9]. Our study found that each child with a depression score that met or exceeded the DSRSC cutoff score for likely clinical depression also met or exceeded the CPSS cutoff score for likely clinical PTSD. These results reflect previous studies that showed adolescents often experience overlapping symptoms of depression and PTSD in a post-disaster setting [9, 40]. The results from the CYRM-12 showed little variation across sociodemographic background and earthquake exposures. Despite a clear need for resilience research in cross-cultural contexts, little is known about how these results compare to others since few studies have explicitly examined resilience. Typically, cultural, religious, sociodemographic, and developmental stage appear to play a role in impacting resilient characteristics in children [11, 41].

Since initial studies focused primarily on the mental health outcomes of adults in Nepal, this study adds to the growing research on the mental health outcomes of Nepali children and adolescents, specifically those in the hard-hit Sindhupalchowk district [18, 42, 43]. Due to the geographical remoteness of the village surveyed, this study also provides some of our first insights into the mental health status of a rural and difficult to reach population. Some of this study’s strengths lie in the variety of respondents who were interviewed and screening tools used for assessment through well-trained translators. The percentage of respondents displaying high resilience in this sample of Nepali children and adolescents adds to the bourgeoning field of research on cultural factors underlying resilient traits in children on a global scale. Studies like this research will help providers better understand how children in underdeveloped nations respond to natural disasters. Understanding response ensures appropriate mental health care can be given in disaster relief settings, as well as during recovery when the affected community starts to rebuild.

Though the relationship between tragedy and mental health problems is clear, the prevalence of adverse outcomes can vary greatly across populations. These wide ranges in prevalence may be attributed to vast cultural and geographic differences, sample characteristics, and the surveys used to measure the severity of mental health problems. Scores can vary greatly for children even from the same country, particularly in countries with remote communities, and inequalities due to ethnic and cultural diversity. There is a need to validate the screening and scoring processes used in various settings, particularly in children, so that results can be more comparable across populations [21, 33, 44]. An additional weakness in this study is the small sample size. With only 62 respondents, the research is not likely to represent the mental health of Nepali children and adolescents as a whole after the 2015 earthquakes.

## Conclusion

In conclusion, this research provided insight to the experiences of children and adolescents in a hard-hit village in Nepal after the 2015 earthquakes. A wider variety of exposures should be taken into account in the future to better understand their relationships to mental health outcomes at the national and global level. While the state of research on mental health in Nepal is in its infancy, additional research is required to inform a more accurate baseline for the mental health status of the country, taking into account its various caste/ethnic groups and economic strata. An association between mental health outcomes in the primary caregiver and the child may also provide more insight into the child’s overall mental health following a natural disaster. Future studies may look into protective factors, such as parent–child attachment and relationship quality, which could contribute to the high level of resilience observed in this sample of Nepali children and adolescents. This research demonstrated potential links between mental health outcomes and both the quantity and type of earthquake exposures experienced by children and adolescents. Therefore, adoption of comprehensive, yet rapid mental health and exposure assessments should be encouraged in short-term and long-term disaster response action plans, especially in areas with less-developed capacities for response.

## Additional file

**Additional file 1.** Earthquake Exposure Assessment Tool for Children and Adolescents.
