# Supplementary material for: Earthquake exposures and mental health outcomes in children and adolescents from Phulpingdanda village, Nepal: a cross-sectional study
Source: Child Adolesc Psychiatry Ment Health. 2018 Dec 20;12:54. doi: 10.1186/s13034-018-0257-9 (PMC6300918; doi:10.1186/s13034-018-0257-9)
Supplement: Supplementary file 1 — Additional file 1. Earthquake Exposure Assessment Tool for Children and Adolescents. [file 13034_2018_257_MOESM1_ESM.docx]

Earthquake Exposure Assessment Tool for Children and Adolescents

**Adapted directly from:* National Child Traumatic Stress Network (September, 2005). Hurricane Assessment and Referral Tool for Children and Adolescents

Child Study ID Number _______________________

DATE ASSESSMENT TOOL ADMINISTERED: ______ / _______ / ____________

RISK CATEGORIES: (CHOOSE ALL THAT APPLY)

| - (1) Child was seriously injured - (2) Family member/friend seriously injured or killed;   Who?___________________   - (3) Witnessed injury/death - (4) Was separated from parent(s) or primary caretaker(s) - (4a) Is currently separated from parents or primary caretaker(s)   With whom is child living at present? _________________   - (5) Home destroyed, badly damaged by earthquakes (circle one) - (5a) Condition of home unknown - (6) Saw neighborhood destroyed or badly damaged - (6a) Saw other areas destroyed or badly damaged - (7) Pet: separated from, lost, hurt or killed (circle one) - (8) Belongings, clothes/toys destroyed by earthquakes - (8a) Condition of belongings unknown - (9) Trapped/difficulty evacuating - (10) Isolated - (11) In other crowded shelter | - (12) Exposed to violence or looting specify:__________________ - (13) Displaced from home; Length of time:____________ - (14) Number of shelter/displacement centers: _________ - (14a) Currently in shelter/displacement center; Length of time: _______ - (15) Transferred to new school because of earthquakes - (15a) Length of time in new school _________ weeks - (15b) Currently out of school because of earthquake _______weeks out of school - (16) Helped in rescue/recovery efforts - (17) Family member served as rescue/recovery worker - (18) Parent unemployed - (18a) Before the earthquakes - (18b) because of earthquakes - (19) Previous earthquake experience - (20) Past major loss or trauma; Briefly describe: __________________________ - (21) Other loss:_____________________________ |
| --- | --- |

**TOTAL NUMBER OF RISK FACTORS IDENTIFIED: (21 items)**
